# Hypoxia-Induced Cisplatin Resistance in Non-Small Cell Lung Cancer Cells Is Mediated by HIF-1α and Mutant p53 and Can Be Overcome by Induction of Oxidative Stress

**DOI:** 10.3390/cancers10040126

**Published:** 2018-04-21

**Authors:** Christophe Deben, Vanessa Deschoolmeester, Jorrit De Waele, Julie Jacobs, Jolien Van den Bossche, An Wouters, Marc Peeters, Christian Rolfo, Evelien Smits, Filip Lardon, Patrick Pauwels

**Affiliations:** 1 Center for Oncological Research (CORE), University of Antwerp, Universiteitsplein 1, 2610 Antwerp, Belgium; vanessa.deschoolmeester@uantwerpen.be (V.D.); jorrit.dewaele@uantwerpen.be (J.D.W.); julie.jacobs@uantwerpen.be (J.J.); jolien.vandenbossche@uantwerpen.be (J.V.d.B.); an.wouters@uantwerpen.be (A.W.); marc.peeters@uza.be (M.P.); evelien.smits@uantwerpen.be (E.S.); filip.lardon@uantwerpen.be (F.L.); Patrick.pauwels@uza.be (P.P.); 2 Department of Pathology, Antwerp University Hospital, Wilrijkstraat 10, 2650 Antwerp, Belgium; 3 Department of Medical Oncology, Antwerp University Hospital, Wilrijkstraat 10, 2650 Antwerp, Belgium; christian.rolfo@uza.be; 4 Phase-1 Early Clinical Trials Unit, Antwerp University Hospital, Wilrijkstraat 10, 2650 Antwerp, Belgium

**Keywords:** p53, HIF-1α, cisplatin resistance, hypoxia, NSCLC

## Abstract

The compound APR-246 (PRIMA-1^MET^) is a known reactivator of (mutant) p53 and inducer of oxidative stress which can sensitize cancer cells to platinum-based chemotherapeutics. However, the effect of a hypoxic tumor environment has been largely overlooked in this interaction. This study focusses on the role of hypoxia-inducible factor-1α (HIF-1α) and the p53 tumor suppressor protein in hypoxia-induced cisplatin resistance in non-small cell lung cancer (NSCLC) cells and the potential of APR-246 to overcome this resistance. We observed that hypoxia-induced cisplatin resistance only occurred in the p53 mutant NCI-H2228^Q331^* cell line, and not in the wild type A549 and mutant NCI-H1975^R273H^ cell lines. Cisplatin reduced HIF-1α protein levels in NCI-H2228^Q331^* cells, leading to a shift in expression from HIF-1α-dependent to p53-dependent transcription targets under hypoxia. APR-246 was able to overcome hypoxia-induced cisplatin resistance in NCI-H2228^Q331^* cells in a synergistic manner without affecting mutant p53^Q331^* transcriptional activity, but significantly depleting total glutathione levels more efficiently under hypoxic conditions. Synergism was dependent on the presence of mutant p53^Q331^* and the induction of reactive oxygen species, with depletion of one or the other leading to loss of synergism. Our data further support the rationale of combining APR-246 with cisplatin in NSCLC, since their synergistic interaction is retained or enforced under hypoxic conditions in the presence of mutant p53.

## 1. Introduction

Lung cancer remains the leading cause of cancer-related deaths worldwide and is characterized by a poor overall survival. Although several new therapeutic strategies are being developed, cisplatin (CDDP)-based therapy remains the golden standard for treatment of late stage non-small cell lung cancer (NSCLC) [1]. Despite a consistent rate of initial responses, cisplatin treatment often results in the development of resistance through various mechanisms, leading to therapy failure and relapse [2,3].

Solid tumors are often characterized by regions with reduced oxygen levels, which can impact the efficacy of cytotoxic drugs. Hypoxia-inducible factor (HIF)-1 is a major regulator in the response to hypoxia by acting as a transcription factor for genes involved in angiogenesis, metabolism, and cell cycle progression, to mediate cellular adaptation to hypoxia [4]. HIF-1 is a heterodimeric complex, consisting of a constitutively expressed β-subunit and an oxygen-regulated α-subunit [5]. HIF-1α has been shown to regulate p53 tumor suppressor protein levels in response to hypoxia, while on the other hand p53 can also negatively regulate HIF-1α protein levels. This complex interaction is strongly dependent on oxygen levels, the duration of hypoxic exposure, and even cell type, as proposed by the model of Sermeus and Michiels [6]. Numerous studies have shown that under mild hypoxic conditions, p53 protein levels are decreased, thereby protecting the cells against apoptosis and promoting cell survival as an adaptive mechanism to the hypoxic environment, a process regulated by HIF-1α. Under severe hypoxic or anoxic conditions, p53 is stabilised, thereby decreasing HIF-1α transcriptional activity, promoting HIF-1α ubiquitination, and inducing apoptotic cell death [6,7]. In addition, HIF-1α and p53 compete for the interaction with CBP (CREB-binding protein) and p300 transcriptional co-activators [6]. The presence of mutant p53 could disrupt this balance, leading to increased levels and activity of HIF-1α and decreased apoptosis in response to hypoxia [8]. As such, high levels of HIF-1α have been reported to negatively influence the response to cisplatin treatment in various tumor types, including lung cancer [9,10,11]. It has been shown that a positive selection pressure exists for p53-deficient (mutant) cells in hypoxic tumor regions, allowing clonal expansion of cells with decreased apoptotic potential, chemoresistant properties, and high metastatic capabilities [6,7,12,13].

In this study, we focused on hypoxia-induced cisplatin resistance, particularly on the interaction between p53 and HIF-1α, to restore treatment response in a panel of NSCLC cell lines with different p53 status using the compound APR-246 (PRIMA-1^MET^). All p53 mutants should not be considered as functionally equivalent, but represent a distinct set of proteins with different properties [14]. Therefore, we included NSCLC cell lines with wild type p53 (A549), a hotspot missense mutation in the DNA binding domain (DBD) (R273H, NCI-H1975) and a nonsense mutation in the oligomerization domain (OD) (Q331*, NCI-H2228) to represent distinct classes of mutant p53. The type of p53 mutation also has implications for the treatment with APR-246. APR-246 is converted to methylene quinuclidinone (MQ), which reacts with thiols in cysteines in the p53 core domain, thereby restoring normal conformation of certain types of mutant p53, including p53^R273H^ [15]. As such, APR-246 is expected to have limited effect on the transcriptional activity of the p53^Q331^* mutant. In addition, mutant p53-independent activity of APR-246 has frequently been reported due to its impact on the cellular redox balance through the inhibition of major antioxidants such as glutathione (GSH) and thioredoxin reductase 1 [16]. Interestingly, hypoxia increased the susceptibility of mutant p53 breast cancer SKBR3 cells to lower PRIMA-1 levels, and synergistic interactions between APR-246 and cisplatin have frequently been reported [16,17]. To the best of our knowledge, we are the first to show that the synergistic interaction between APR-246 and cisplatin is retained or enhanced under hypoxic conditions in a p53-mutant background. In addition, we show that APR-246 can overcome hypoxia-induced cisplatin resistance in an ROS-dependent and mutant p53^Q331^*-dependent manner through GSH depletion rather than reactivation of mutant p53 in the NCI-H2228 NSCLC cell line.

## 2. Results

### 2.1. APR-246 Acted Synergistically with Cisplatin in a Mutant p53 Background under Normal and Reduced Oxygen Levels

**Figure 1:**
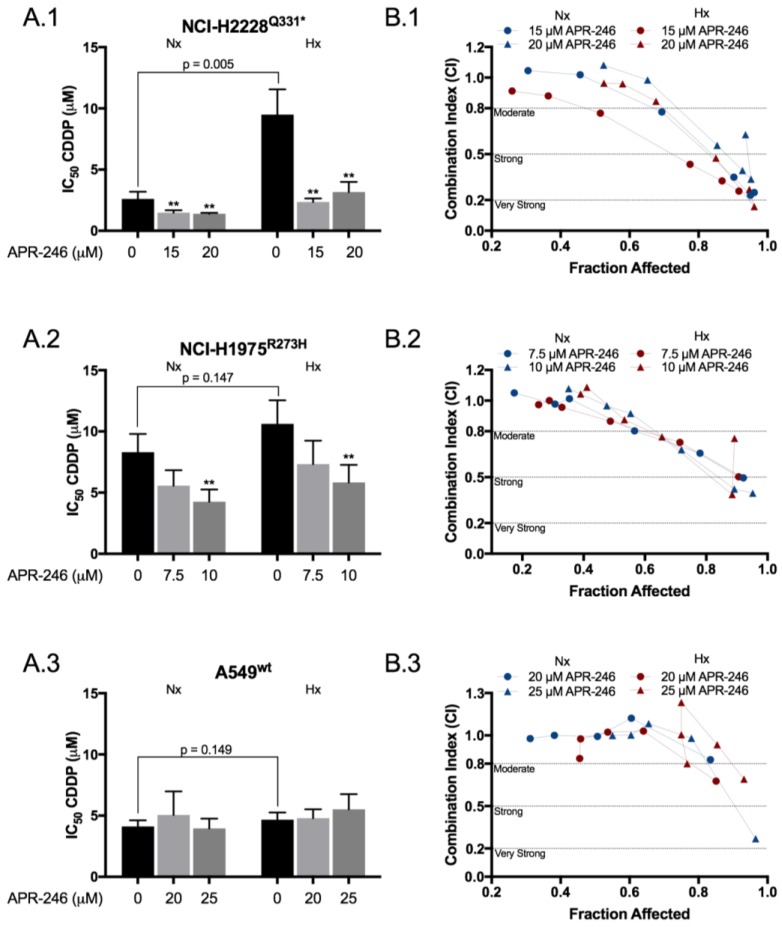
APR-246 synergistically enhanced the cytotoxic effect of CDDP in a p53-mutant background under both normoxic and hypoxic conditions. (**A1**–**A3**) To determine possible synergism, cells were simultaneously treated for 72 h with a CDDP (cisplatin) dose range of 0, 0.5, 1, 2, 5, 10 μM (A549^wt^) or 0, 0.5, 1, 2, 5, 10, 20 μM (NCI-H2228^Q331^* and NCI-H1975^R273H^) and fixed concentrations of APR-246, based on the drug sensitivity of each cell line (Table S1). Cell confluence was determined using the SRB-assay and IC_50_ values were calculated using WinNonlin. IC_50_ values for CDDP monotherapy or combined with APR-246 are presented as mean ± SD of at least 3 independent experiments. (**B1**–**B3**) The corresponding combination index was calculated using the ‘Additive Model’ for each concentration presented, in correlation with the affected fraction (FA) of the cells. CI = 1.0 ± 0.2 indicates an additive effect, <0.8 indicates moderate synergism, <0.5 strong synergism, and <0.2 very strong synergism (** *p* < 0.05 compared to CDDP monotherapy). The corresponding values are presented in Table S1. Nx: normoxia; Hx: hypoxia.

The cytotoxic effects of APR-246 and CDDP were assessed in a panel of three NSCLC cell lines under normoxic and hypoxic conditions using the SRB assay. APR-246 significantly increased the cytotoxic effect of CDDP in the p53 mutant cell lines, as presented by the reduction in IC_50_ values (Figure 1A.1–3 and Table S1). The combination index indicated a moderate to very strong synergistic effect in the p53-mutant cell lines NCI-H1975^R273H^ and NCI-H2228^Q331^*, which was enforced by reduced oxygen conditions in the latter (Figure 1B1,B2). This effect was absent in the p53 wild type cell line A549^wt^ (Figure 1B3). Interestingly, hypoxia-induced CDDP resistance was limited to NCI-H2228^Q331^*, with a nearly 4-fold increase in IC_50_ value (2.61 ± 0.59 vs. 9.49 ± 2.07 µM, *p* = 0.005), which was restored to normoxic values following co-treatment with APR-246 (Figure 1A1, Table S1).

### 2.2. Hypoxia Affected the Apoptotic Response to CDDP and APR-246 Monotherapy, While Co-Treatment Resulted in an Equally Strong Apoptotic Response Compared to Normoxia in NCI-H2228^Q331^*

**Figure 2:**
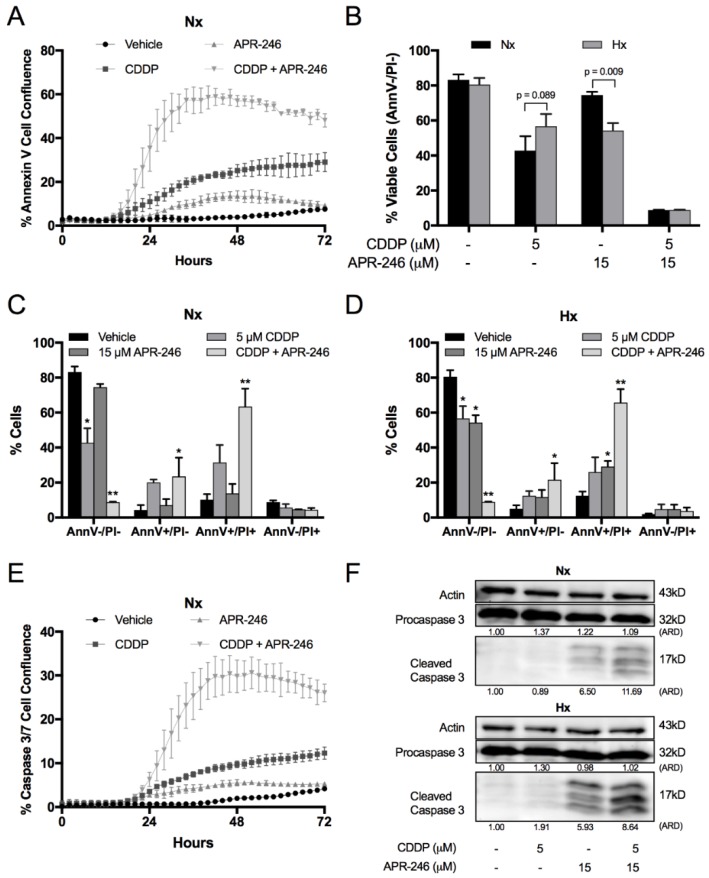
Influence of oxygen levels and treatment on apoptosis of NCI-H2228 cells. Cells were treated for 72 h (**A**–**E**) or 24 h (**F**) with vehicle, CDDP, APR-246, or CDDP/APR-246. (**A**) Apoptosis was monitored using the IncuCyte Annexin V Green reagent under normoxic conditions. Presented as mean ± SD Green Object Confluence/Phase Object Confluence of 3 replicates. (**B**) Viability was assessed by flow cytometry using the AnnexinV/PI assay. AnnV-/PI-cells are marked as viable cells. Data is presented as mean ± SD of 3 independent experiments. (**C**,**D**) Apoptosis/cell death was assessed by flow cytometry under normoxic and hypoxic conditions. Percentage of AnnV−/PI−; AnnV+/PI−; AnnV+/PI+ and AnnV−/PI+ cells is shown as mean ± SD of 3 independent experiments. * *p* < 0.05 compared to vehicle treated sample; ** *p* < 0.05 compared to vehicle, CDDP and APR-246 treated samples. Dotplots are presented in Figure S1. (**E**) Caspase activity was monitored using the IncuCyte Caspase-3/7 Green reagent. Presented as mean ± SD of 3 replicates Green Object Confluence/Phase Object Confluence. (**F**) Procaspase-3 and cleaved caspase-3 levels were determined by Western blotting; β-actin was used as an internal standard. ARD: Adjusted Relative Density; Nx: normoxia; Hx: hypoxia.

We focussed in depth on the apoptotic response in NCI-H2228^Q331^*, since this was the only cell line showing hypoxia-induced CDDP resistance as well as the strongest synergistic effect of the combination therapy. Apoptosis was initiated within the first 24 h of treatment (Figure 2A), and co-treatment ultimately killed nearly all tumor cells after 72 h under both normoxic (Figure 2B and Figure S1) and hypoxic (Figure 2D and Figure S1) conditions. Hypoxia reduced the cytotoxic response to CDDP, but increased this response to APR-246, while no difference was observed after co-treatment compared to normoxia (Figure 2B). Next, we monitored caspase 3/7 activity in real time (Figure 2E), which was initiated within 24 h after initiation of the treatment under normoxic conditions. At 24 h, it was clearly observed that hypoxia strongly enforced caspase 3 cleavage after APR-246 and co-treatment compared to normoxic conditions (Figure 2F), indicating an earlier and stronger apoptotic response to APR-246 and co-treatment under hypoxia.

### 2.3. CDDP Induced a Shift from HIF-1α to p53^Q331^* Protein Expression and Transcription under Both Normoxic and Hypoxic Conditions

**Figure 3:**
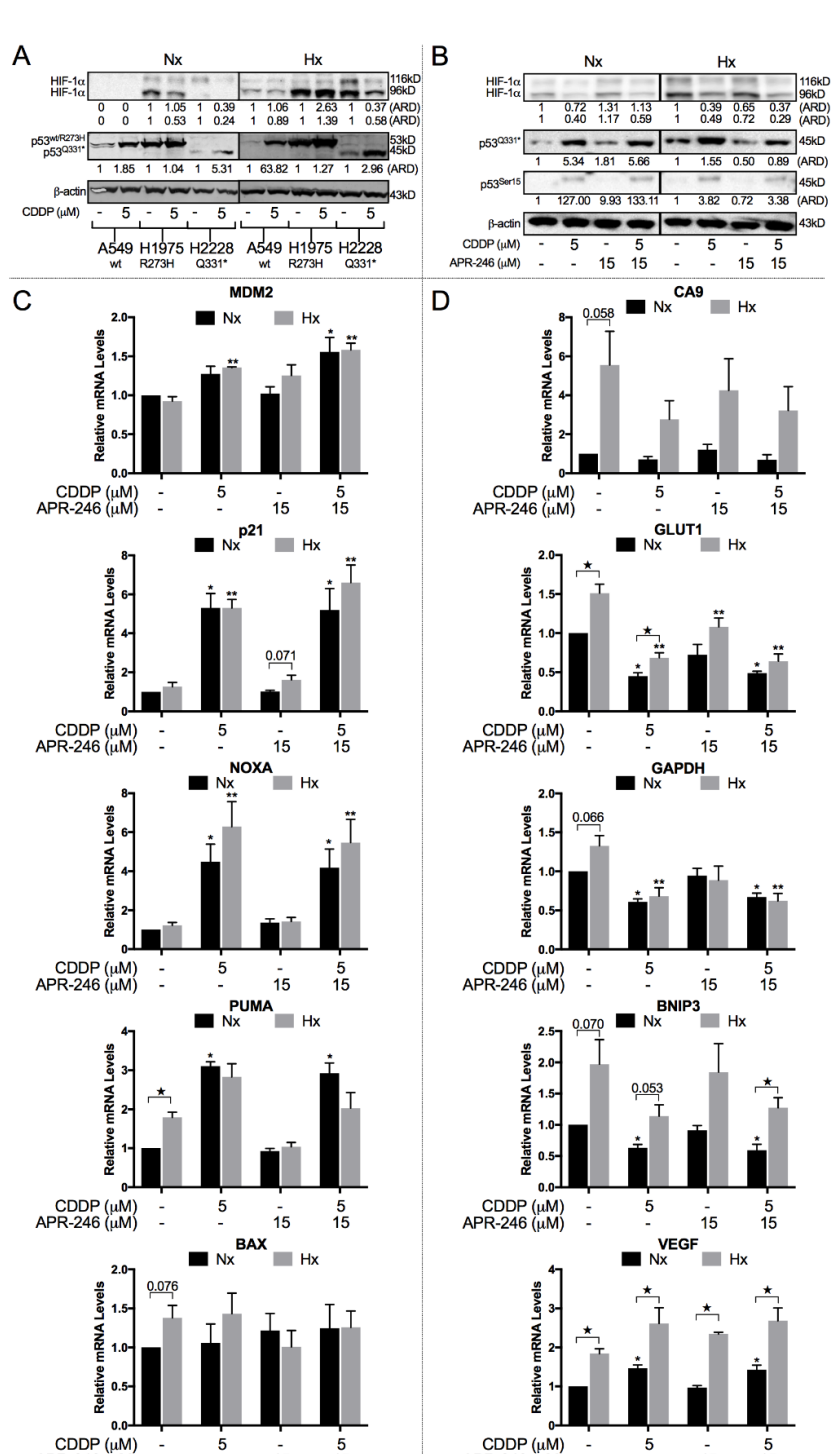
Balance between HIF-1α and p53 protein levels and transcriptional activity. (**A**) A549^wt^, NCI-H1975^R273H^, and NCI-H2228^Q331^* cell lines were treated with either vehicle or CDDP under normoxic and hypoxic conditions. Protein levels of HIF-1α 116 kDa (processed), HIF-1α 96 kDa (unprocessed), full p53^wt/R273H^, and truncated p53^Q331^* were determined by Western blotting. β-actin was used as internal control. (**B**–**D**) NCI-H2228 cells were treated for 24 h with with CDDP, APR-246, or combined therapy under normoxic and hypoxic conditions. (**B**) Protein levels of HIF-1α 116 kDa (processed), HIF-1α 96 kDa (unprocessed), p53^Q331^*, and p53-serine 15 were determined by Western blotting. β-actin was used as internal control. (**C**,**D**) Relative mRNA expression levels of p53 (**C**) and HIF-1α (**D**) transcriptional targets. Expression levels are presented relative to the vehicle-treated sample under normoxia as mean ± SEM of 3 independent experiments (★ *p* < 0.05 normoxia vs. hypoxia; * *p* < 0.05 compared to vehicle-treated sample under normoxia; ** *p* < 0.05 compared to vehicle-treated sample under hypoxia.). ARD: Adjusted Relative Density; Nx: normoxia; Hx: hypoxia.

Next, we determined the role of p53 and HIF-1α in hypoxia-induced CDDP resistance. Both NCI-H1975^R273H^ and NCI-H2228^Q331^* cell lines showed detectable levels of HIF-1α under normoxic conditions, which were further induced under hypoxia (Figure 3A). In A549^wt^, HIF-1α was only detected under hypoxic conditions (Figure 3A). Interestingly, HIF-1α protein levels were strongly reduced under both normoxic and hypoxic conditions in response to CDDP therapy in NCI-H2228^Q331^*, which was the only cell line characterized by hypoxia-induced CDDP resistance (Figure 3A,B). In NCI-H1975^R273H^, a reduction in HIF-1α protein levels after CDDP treatment was observed under normoxic conditions. However, this effect was not retained under hypoxic conditions (Figure 3A). Mutant p53 protein levels were slightly increased in response to hypoxia, and both mutant and wild type p53 levels were increased in response to CDDP treatment under both normoxic and hypoxic conditions (Figure 3A).

NCI-H2228^Q331^* cells showed an increase in p53 protein levels, including transcriptionally active p53Ser15, and a clear reduction in HIF-1α protein levels in response to CDDP treatment, suggesting a potential switch in transcriptional activity (Figure 3B). A clear increase in p53 transcription targets (p21, NOXA, PUMA, and MDM2) was observed in response to CDDP (Figure 3C). As expected, all HIF-1α transcription targets (CA9, GLUT1, GAPDH, BNIP3, and VEGF) were increased in response to hypoxia (Figure 3D). All HIF-1α targets, except for VEGF, were reduced in response to CDDP treatment in NCI-H2228^Q331^* cells. APR-246 had limited to no effect on p53, p53Ser15 protein levels, and the expression of p53 transcription targets.

**Figure 4:**
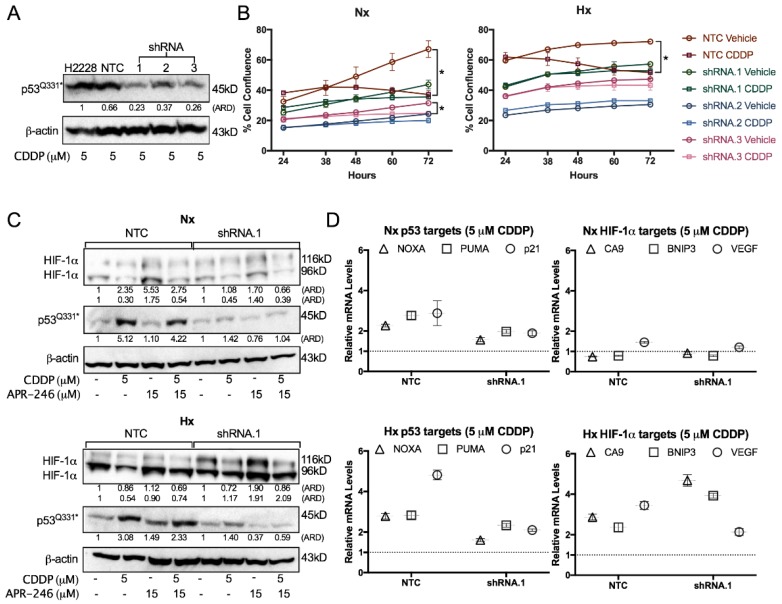
Role of p53 in CDDP resistance. (**A**) P53 protein levels determined by Western blotting after 48 h treatment with 5 µM CDDP of the NCI-H2228^Q331^* parental cell line, nontemplate control (NTC), and three *TP53* shRNA monoclonal subclones under normoxia. β-actin was used as an internal standard. (**B**) Cell proliferation presented as mean ± SD of 3 replicates Green Object Confluence using the IncuCyte system. Cells were treated with either vehicle or 5 µM CDDP under normoxia or 1% O_2_. (**C**) Protein levels of HIF-1α 116 kDa (processed), HIF-1α 96 kDa (unprocessed), and p53 were determined by Western blotting after treatment with vehicle, CDDP, APR-246, or combination therapy for 24 h in NTC and shRNA.1 (*TP53* shRNA) cell lines under normoxic and hypoxic conditions. β-actin was used as internal control. (**D**) mRNA levels of p53 (NOXA, PUMA, p21) and HIF-1α (CA9, BNIP3, VEGF) transcription targets. NTC and shRNA.1 cells were treated with 5 µM CDDP for 24 h under normoxia or 1% O_2_ and expression levels are presented relative to the vehicle-treated sample under normoxia. (* *p* < 0.05 compared to vehicle-treated sample at the 72 h time-point). ARD: Adjusted Relative Density; Nx: normoxia; Hx: hypoxia.

To study the potential role of mutant p53^Q331^* in HIF-1α downregulation and the expression of p53 transcription targets, we stably transfected NCI-H2228^Q331^* cells with a set of three vectors containing *TP53* shRNA, in order to knock down mutant p53 levels (Figure 4A). Mutant p53 was clearly a strong mediator of the cytotoxic response to CDDP under both normoxic and hypoxic conditions, as indicated by the absence of confluence reduction in the p53 knockdown clones compared to the nontemplate control (NTC) (Figure 4B). When looking at unprocessed HIF-1α protein levels (96 kDa) under hypoxic conditions, a clear reduction was observed after treatment with CDDP in NTC cells, while this reduction was absent in shRNA.1 cells, indicating that p53^Q331^* is involved in the CDDP-dependent reduction of HIF-1α (96 kDa). In addition, p53 knockdown led to a strong increase in processed HIF-1α protein levels (116 kDa) compared to NTC, while a similar trend of p53 increase and HIF-1α downregulation in response to CDDP were still observed compared to the parental cell line due to the residual p53 protein in shRNA.1 (Figure 4C). Furthermore, CDDP-induced expression of NOXA, PUMA, and p21 was reduced after p53 knockdown under both normoxic and hypoxic conditions, suggesting that transcription of these target genes is p53^Q331^*-dependent (Figure 4D). For the HIF-1α transcriptional targets CA9, BNIP3, and VEGF, we did not observe any change in expression under normoxic conditions. On the other hand, higher levels of CA9 and BNIP3 were observed after CDDP treatment in the p53 knockdown clone compared to the NTC clone, while lower levels of VEGF were observed, indicating increased HIF-1α-dependent transcription (Figure 4D).

Overall, we observed that CDDP reduced HIF-1α protein levels as well as mRNA expression levels of several of its transcription targets, while at the same time an increase in p53 protein levels and transcription targets was observed. In addition, all these effects were mutant p53^Q331^*-dependent as they were affected by p53 knockdown.

### 2.4. The Synergistic Interaction between APR-246 and CDDP was Both Mutant p53^Q331^*- and ROS-Dependent Following APR-246-Induced GSH Reduction under Hypoxic Conditions

**Figure 5:**
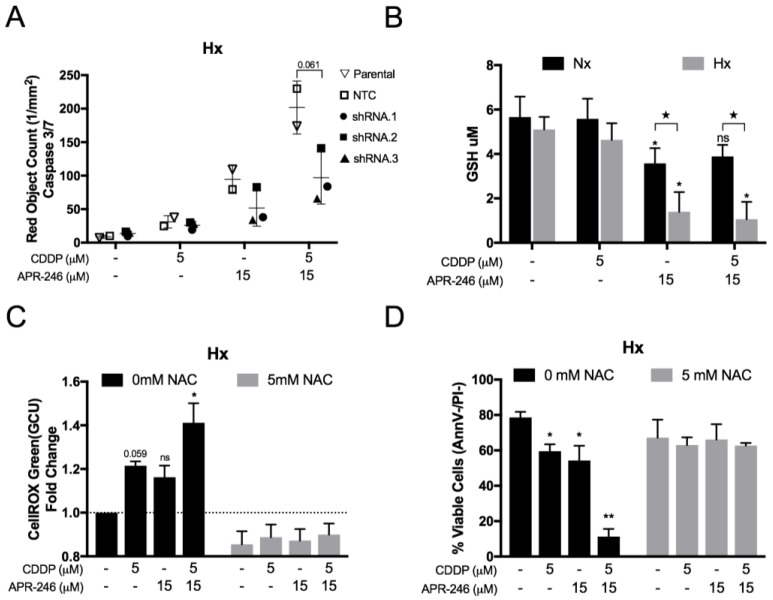
Role of p53^Q331^* and ROS in the synergistic combinatory effect of CDDP and APR-246 under hypoxia. (**A**) NCI-H2228^Q331^* parental cell line, non-template control (NTC), and three *TP53* shRNA monoclonal subclones were treated with APR-246 or CDDP/APR-246 under 1% O_2_. Caspase-3/7 activity was measured using the IncuCyte Caspase 3/7 Red reagent under hypoxia and presented as mean ± SD of the number of Red Object Counts/mm^2^ of 3 replicates. (**B**) NCI-H2228^Q331^* cells were treated with vehicle, CDDP, APR-246, or CDDP/APR-246 for 24 h and total glutathione (GSH) content was measured under normoxia and hypoxia. Data is presented as mean µM GSH/well ± SD of 3 independent experiments. (**C**) NCI-H2228^Q331^* cells were treated with vehicle, CDDP, APR-246, or CDDP/APR-246 for 72 h in the absence or presence of 5 mM NAC under 1% O_2_. Intracellular mean intensity (GCU) of the CellROX dye was measured using the IncuCyte system and presented as mean fold change to untreated sample ± SEM of 4 independent experiments (**D**) NCI-H2228 cells were treated with vehicle, CDDP, APR-246, or CDDP/APR-246 for 72 h in the absence or presence of 5 mM NAC under 1% O_2_. The percentage of viable cells (AnnV−/PI−) was measured by flow cytometry and presented as mean percentage ± SD of 3 independent experiments. (★ *p* < 0.05 Nx vs. Hx; * *p* < 0.05 compared to vehicle-treated sample; ** *p* < 0.05 compared to vehicle-, CDDP-, and APR-246-treated samples) Nx: normoxia; Hx: hypoxia.

As indicated by the data presented in Figure 3, it is clear that APR-246 did not affect p53^Q331^* protein levels, nor did it affect p53^Q331^* transcriptional activity for the selected targets, either as monotherapy or in combination with CDDP. Therefore, the hypothesis that APR-246 could induce a shift from HIF-1α- to p53-dependent transcriptional activity was not supported. Nevertheless, p53 knockdown reduced the apoptotic response to APR-246 and greatly reduced the synergistic apoptotic effect after co-treatment with CDDP under normoxic conditions as indicated by the reduction in caspase 3/7 positive cells in p53 knockdown cell lines (shRNA.1–3) versus the NTC and parental cell line (Figure 5A). This reduction was consistent with the residual p53 levels, as shRNA.2 cells had the highest residual p53 levels and highest level of caspase 3/7 positive cells compared to shRNA.1 and shRNA.3 (Figure 4A).

Next, we measured the total cellular GSH content after the different treatment options. While cisplatin did not affect GSH levels, a significant reduction was observed after APR-246 treatment under both normoxic and hypoxic conditions. Interestingly, APR-246 GSH inhibition was significantly more effective under hypoxic conditions (Figure 5B).

Both CDDP and APR-246 increased cellular ROS levels, as indicated by a noticeable increase in CellRox intensity (GCU), while a stronger and significant increase was observed after combined treatment under hypoxia (Figure 5C). Using the ROS scavenger NAC, we were able to reduce basal and therapy-induced ROS accumulation in the NCI-H2228^Q331^* cells (Figure 5C). In addition, NAC completely annulled the synergistic apoptotic response to APR-246 and CDDP co-treatment under hypoxia as indicated by the number of viable (AnnV−/PI−) cells (Figure 5D). Furthermore, NAC treatment inhibited the cytotoxic effect of both CDDP and APR-246 as monotherapy, indicating a prominent role of ROS induction for both compounds under hypoxia. In conclusion, both the presence of mutant p53^Q331^* and ROS accumulation seem crucial mediators in the synergistic interaction between APR-246 and CDDP under hypoxia. 

## 3. Discussion

The findings presented in this study support our hypothesis that APR-246 can be used to overcome hypoxia-induced cisplatin resistance in vitro in NCI-H2228^Q331^* cells. Numerous studies have previously reported strong synergistic effects for the combination of cisplatin with APR-246 [18,19,20], consistent with our results under normoxic conditions in a p53-mutant background. To the best of our knowledge, our study is the first to show that APR-246 more efficiently inhibits GSH under hypoxic conditions and is able to overcome hypoxia-dependent resistance to cisplatin in a mutant p53- and ROS-dependent manner. In addition, we are the first to show that cisplatin can act on HIF-1α protein levels through the induction of truncated mutant p53^Q331^*, leading to a shift from HIF-1α- to p53-dependent transcription. A limitation of this study is that the data is supported by one cell line. We searched the COSMIC Cell Lines Project v83 and IARC database for NSCLC cell lines with a similar truncating mutation in the OD, but no such cell line was found. 

Hypoxia-induced cisplatin resistance was limited to the NCI-H2228^Q331^* cell line. Interestingly, this was the only tested NSCLC cell line in which cisplatin reduced HIF-1α protein levels under hypoxic conditions. The fact that cisplatin did not affect HIF-1α levels in the p53 wt A549 cell line is in line with findings previously reported by others [21]. The role of wild type p53 in the regulation of HIF-1α has extensively been discussed by others, suggesting that DNA damage is a prerequisite for p53-mediated repression of HIF-1α [6,22]. However, this was not observed by us in A549^wt^ cells in response to cisplatin treatment, indicating that other factors might be at play.

It should be noted that there is a clear difference in the p53 mutations of both mutant cell lines used in this study. The R273H mutation in the NCI-H1975^R273H^ cell line is a hotspot mutation in the p53 DBD, while the NCI-H2228^Q331^* harbors a truncating mutation in the p53 OD, suggesting that different types of mutations might have different effects on HIF-1α. Our observations warrant further investigation into the effects of similar truncating mutations, whether or not in the p53 OD. Although less frequent, truncating mutations still account for 36% of the p53 mutations found in the TCGA LUAD (The Cancer Genome Atlas, Lung Adenocarcinoma) cohort, of which 12% occurs in the OD [23].

Similar to Mohell et al., we observed strong synergistic effects when combining CDDP with APR-246 in the NCI-H1975^R273H^ cells under normoxic conditions [19]. They proposed activation of p53 transcription as a potential cause of synergism, which we did not observe in our study in the NCI-H2228^Q331^* cell line. Hypoxia did not enforce the synergistic cytotoxic effect in NCI-H1975^R273H^ cells, in contrast to the NCI-H2228^Q331^* cell line. Our initial hypothesis that APR-246 could induce a shift from HIF-1α-dependent transcription, promoting cell survival, towards p53-dependent transcription, promoting cell death/apoptosis, was not supported by our data. However, we did observe that cell death following the combination therapy was dependent on both the presence of mutant p53^Q331^* and the induction of ROS, with inhibition of one or the other leading to the loss of synergism. In addition, we showed that cisplatin induced mutant p53^Q331^*-dependent HIF-1α degradation. Ai et al. recently showed that cisplatin induced HIF-1α degradation in cisplatin-sensitive ovarian cancer cells, but did not downregulate HIF-1α in their cisplatin-resistant counterpart, indicating that HIF-1α downregulation is an important mediator in the response to cisplatin [11]. In addition, knockdown of HIF-1α redirected aerobic glycolysis towards mitochondrial oxidative phosphorylation and enhanced cisplatin-induced cell death through overproduction of ROS as a by-product of oxidative phosphorylation [11]. It should be noted that this study was not performed in the context of hypoxia-induced HIF-1α and did not take into account the p53 status of the different cell lines.

It has been shown that HIF-1α can be upregulated in an ROS-dependent manner to maintain ROS homeostasis by redirecting the cellular metabolism [24,25,26,27]. We observed that HIF-1α protein levels were upregulated in response to APR-246 in p53 knockdown cells, while no noticeable difference was observed in the parental tumor cells. In addition, co-treatment with the ROS scavenger NAC reduced HIF-1α levels under hypoxia in response to cisplatin treatment (data not shown) [28]. These observations indicate that HIF-1α is upregulated in response to oxidative stress under hypoxia. The fact that NAC inhibited the cytotoxic response to cisplatin in our study under hypoxic conditions indicates that ROS has at least a partial role in the cytotoxic response. 

**Figure 6:**
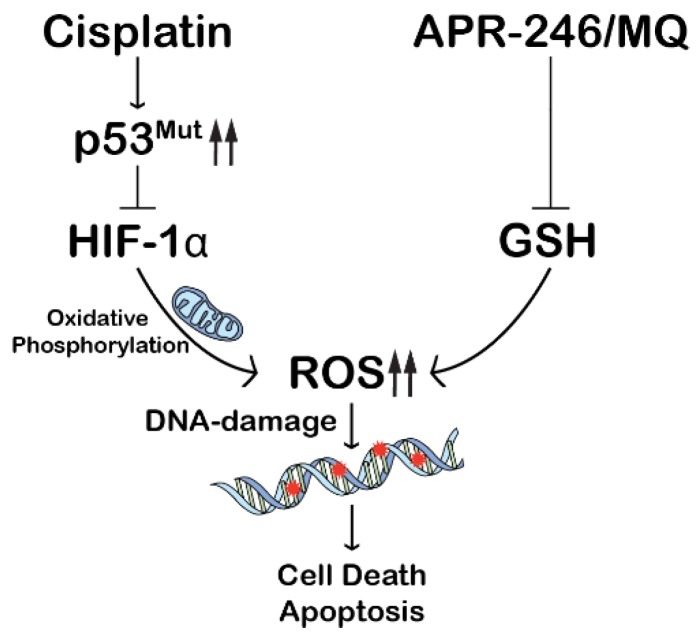
Underlying mechanism of the synergistic interaction between CDDP and APR-246 under hypoxic conditions. Cisplatin induces upregulation of mutant, truncated p53, which in turn degrades HIF-1α protein. HIF-1α reduction leads to a shift in metabolism, resulting in induction of reactive oxygen species (ROS). APR-246 further increases oxidative stress through inhibition of glutathione (GSH) leading to a synergistic induction of intracellular ROS, which in turn induces massive cell death.

Based on these data we propose the following mechanism of strong synergism in the NCI-H2228^Q331^* cell line under hypoxic conditions (Figure 6): cisplatin induces upregulation of mutant, truncated p53, which in turn depletes HIF-1α protein levels. HIF-1α reduction leads to a shift in metabolism, resulting in induction of ROS. By adding APR-246, which effectively depletes cellular GSH content, the antioxidant mechanism is disturbed, leading to a strong synergistic induction of intracellular ROS, which in turn induces massive cell death.

APR-246-dependent GSH depletion is an important mediator of the synergistic antitumor effects we observed and inhibitors of GSH synthesis like buthionine sulfoximine (BSO) could yield similar results. PRIMA-1 and APR-246 have been shown to synergize with BSO, while BSO by itself did not affect cell viability in MCF-7 tumor cells [17,29,30]. The additional mechanisms of action of APR-246, like reactivation of certain forms of mutant p53 or induction of ER stress, can affect tumor cells in various ways resulting in an enhanced antitumor efficacy compared to BSO. 

## 4. Materials and Methods

### 4.1. Cell Culture

The NSCLC cell lines A549^wt^ (CCL-185, TP53^wt^), NCI-H1975^R273H^ (CRL-5908, TP53^R273H^), and NCI-H2228^Q331^* (CRL-5935, TP53^Q331^*) were obtained from ATCC. Cells were grown as monolayers and maintained in exponential growth in 5% CO_2_/95% air in a humidified incubator at 37 °C under normoxic conditions. A549^wt^ cells were cultured in DMEM (10% FBS, 100 U/mL penicillin, 100 µg/mL streptomycin, and 2 mM l-glutamine) (Life Technologies, Carlsbad, CA, USA). NCI-H1975^R273H^ and NCI-H2228^Q331^* were cultured in RPMI supplemented as described above, with the addition of 1 mM sodium pyruvate (Life Technologies).

Experiments performed under reduced oxygen conditions were plated out and settled overnight under normoxic conditions as described above. A Bactron IV anaerobic chamber (Shell Lab) was used to obtain hypoxic conditions (1% O_2_; 4% CO_2_; 95% N_2_). Cells were adjusted to reduced oxygen levels for 6 h before treatment was initiated, after which HIF-1α protein levels were increased (Figure S2).

For APR-246 (PRIMA-1^MET^; Tocris, Bristol, UK) a stock solution of 50 mmol/L was prepared in dimethyl sulfoxide (DMSO) and stored at −20 °C. For cisplatin (CDDP, Tocris), a stock solution of 5 mmol/L was prepared in 0.9 NaCl and stored at −80 °C. Dilutions were made in PBS.

### 4.2. Survival Assay and Synergism

End-point cell density was assessed using the sulforhodamine B (SRB) assay as previously described [31]. To determine IC_50_ values, cells were exposed to 0–20 μM cisplatin or 0–40 μM APR-246 for 72 h and IC_50_ values were calculated using WinNonlin^®^ software (Phoenix, AZ, USA). Combination studies were performed by simultaneously exposing cells to a concentration range of CDDP (0–20 μM) and a fixed concentration of APR-246, corresponding with approximately IC_20_ or IC_40_ values for each cell line for 72 h. In order to determine possible synergism, data was analysed according to the ‘Additive Model’, as described by others [19,32,33]. All experiments were performed under both normoxic and hypoxic conditions.

### 4.3. Western Blot Analysis

Cells were treated with either vehicle, a fixed cell line-dependent concentration of CDDP, APR-246, or CDDP/APR-246 combined treatment for 24 h under normoxic and hypoxic conditions. Western blotting was performed as previously described [34]. Blocking and primary and secondary antibody incubation were performed using the SNAP id^®^ 2.0 protein detection system (Merck Millipore, Burlington, MA, USA) according to the manufacturer’s instructions. The following antibodies were used: rabbit monoclonal anti-p53 (Cell Signalling Technology, Danvers, MA, USA, no. 9282; 1:1000); rabbit polyclonal anti-phospho-p53 (S15, R&D systems, #AF1043, 1:1000); mouse monoclonal anti-HIF-1α (Becton Dickinson Pharmingen, clone 54/HIF-1α; 1:1000, 96 kDa: unprocessed; 116 kDa: fully post-translationally modified); Apoptosis Western Blot Cocktail (procaspase 3, cleaved caspase 3, β-actin; Abcam no. ab136812); mouse monoclonal anti-β-actin (Sigma-Aldrich, St. Louis, MO, USA; clone AC-15; 1:2500); anti-mouse and anti-rabbit HRP-labelled secondary antibodies (Cell Signalling; no. 7076S and no. 7074S, respectively; 1:2500). Chemiluminescent detection was performed using the Luminata^TM^ Forte Western HRP Substrate (Merck Millipore). Image J software was used to quantify the relative density of the Western Blot bands and was presented as Adjusted Relative Density (ARD): (area sample/area standard)/(area loading control sample/area loading control standard).

### 4.4. Apoptotic/Cell Death Assays

Tumor cells were treated with either vehicle, CDDP, APR-246, or a combination of both under normoxic and hypoxic conditions. Apoptotic cell death was assessed by the Annexin V-FITC/Propidium Iodide assay (Becton Dickinson Pharmingen) after 72 h. All assays were performed on a FACScan flow cytometer (Becton Dickinson) and analysed with FlowJo v10 (TreeStar Inc., Ashland, OR, USA).

Real-time cell proliferation and induction of cell death were monitored using the IncuCyte^®^ ZOOM live-cell analysis instrument (Essen Bioscience, Ann Arbor, MI, USA) equipped with a 10× objective. Cells were plated on a Corning^®^ 96-well plate (Sigma Aldrich) and settled overnight. Images were taken every two hours under normoxic conditions from the start of the treatment. A scan on demand (SCOD) was performed for plates incubated under hypoxic conditions at multiple time points after start of the treatment. To ensure hypoxic conditions were maintained during the SCOD, plates were sealed with an adhesive plate seal inside the hypoxic chamber, protecting the inside of the wells from the normoxic environment in which the IncuCyte^®^ was placed. After each SCOD, the plate was replaced in the hypoxic chamber and the seal was removed. Green channel acquisition time was 400 ms, whereas red channel acquisition time was 800 ms. IncuCyte^®^ reagents (Essen Bioscience) were added at the start of the treatment, after which the image acquisition was started. The Annexin V Green Reagent (Essen Bioscience, Cat #4642) was used at a final dilution of 1:200 in full medium supplemented with 1 mM CaCl_2_. The Caspase 3/7 Green Apoptosis Reagent (Essen Bioscience, Cat #4440) was used at a final concentration of 2.5 µM and the Caspase 3/7 Red Reagent (Essen Bioscience, Cat #4704) at 0.25 µM. Analysis was performed using the IncuCyte^®^ ZOOM2016B software (Ann Arbor, MI, USA). Green and red channel background noise was subtracted with the Top-Hat method. Data are presented either as confluence or object count and normalization is described in the corresponding figure legends.

### 4.5. Quantitative Reverse Transcription PCR (RT-qPCR)

NCI-H2228^Q331^* cells were treated with either vehicle, cisplatin, APR-246, or a combination of both under normoxic and hypoxic conditions. RNA was isolated 24 h post treatment using the TRIzol^®^ method (Life Technologies). Total RNA yield and quality were measured using the NanoDrop^®^ ND-1000 (Thermo Scientific) and stored at −80 °C. RT-qPCR was performed as previously described [35]. A panel of 8 potential housekeeping genes (HKGs) (SDHA1, HMBS, GAPDH, YWHAZ, PMM1, RPL13A, B2M, and HPRT1) were tested on 8 representative samples and the qbase+ software (Biogazelle, Zwijnaarde, Belgium) was used to determine the most stable HKGs. B2M and HPRT1 were selected as optimal reference targets (geNorm M < 0.5 and geNorm V < 0.15). P21, BAX, PUMA, NOXA, and MDM2 were used as genes of interest to assess p53-mediated transcription. CA9, GLUT1, GAPDH, BNIP3, and VEGF were uses as genes of interest to assess HIF-1α-mediated transcription. Primer sequences are presented in Table S2. 

### 4.6. Reactive Oxygen Species Detection

Tumor cells were treated with either vehicle, CDDP, APR-246, or a combination of both under normoxic and hypoxic conditions for 72 h. The CellROX^®^ Green Reagent (Thermo Scientific) was used to monitor oxidative stress in real time using the IncuCyte^®^ ZOOM. The cell-permeant dye is weakly fluorescent while in a reduced state and exhibits bright green photostable fluorescence upon oxidation by reactive oxygen species (ROS) and subsequent binding to DNA. This way, the dye accumulated in the nucleus of the cell in accordance with intracellular basal and induced ROS levels. NCI-H2228^Q331^* cells were treated with either vehicle, cisplatin, APR-246, or a combination of both under hypoxic conditions in the presence of 2.5 µM CellROX by a SCOD under hypoxic conditions at multiple time points. Analysis was performed using the IncuCyte^®^ ZOOM2016B software. Green channel background noise was subtracted with the Top-Hat method. The data are presented as Average Mean Green Intensity (GCU). As a control, cells were co-treated with the ROS scavenger *N*-acetyl-l-cysteine (NAC, Sigma-Aldrich), which acts as a precursor of GSH.

### 4.7. Total Glutathione Content

Tumor cells were treated with either vehicle, CDDP, APR-246, or a combination of both under normoxic and hypoxic conditions for 24 h. Total GSH content was measured with the luminescence-based GSH/GSSG^TM^ assay (Promega, Madison, WI, USA) according to the manufacturer instructions. The luminescent signal was measured using the VICTOR^TM^ X5 Multilabel Plate reader (PerkinElmer, Zaventem, Belgium). Absolute quantification was obtained with a GSH standard curve and concentrations are presented as µM GSH/well.

### 4.8. Transduction

NCI-H2228^Q331^* cells were transduced using the GIPZ lentiviral shRNA VGH5526-EG7157 viral particle set (Thermo Scientific), which consists of one nontemplate control (NTC) vector and three different *TP53* shRNA constructs containing a puromycin resistance gene and green fluorescent protein (GFP) reporter gene. Transduction was performed according to the manufacturer’s instruction with either the NTC vector or three *TP53* shRNA vectors. Selection was made by exposing cells to 5 µg/mL puromycin and monitoring GFP expression. After one week of puromycin selection, single cell colonies were grown for the *TP53* shRNA cells, resulting in three monoclonal subclones, shRNA.1, shRNA.2, and shRNA.3, with stable p53 knockdown as confirmed by Western Blotting. In order to maintain stably transduced cell lines, cells were frequently challenged with puromycin (5 µg/mL). 

### 4.9. Statistical Analysis

Prism 7.03 software (GraphPad, La Jolla, CA, USA) was used for data comparison and artwork. Statistical analysis was performed using SPSS Statistics 24 software (IBM, Armonk, NY, USA). Normality of the data was assessed by the Shapiro–Wilk test. Data that followed a normal distribution (*p* > 0.05) were assessed by unpaired parametric tests to compare medians between two (Unpaired Student *t*-test) or multiple groups (One Way Anova with Tukey post-hoc testing).

## 5. Conclusions

In this study, we show that hypoxia-induced cisplatin resistance only occurred when cisplatin affected HIF-1α protein levels. We showed that HIF-1α protein levels were downregulated in a mutant-p53-dependent manner, more specifically a truncating mutation in the p53 OD, whose expression was induced by cisplatin. This could indicate a novel gain-of-function effect which warrants further investigation of similar (truncating) mutations. Cell death following the combination of APR-246 and cisplatin was dependent on both the presence of mutant p53 and the induction ROS, with inhibition of one or the other leading to the loss of synergism. This ROS accumulation was potentially mediated by a shift in HIF-1α-regulated metabolism due to cisplatin-induced HIF-1α degradation via mutant p53^Q331^* and inhibition of antioxidant GSH by APR-246. Our results further support the use of APR-246 in combination with cisplatin-based therapies in NSCLC patients due to the strong synergistic interaction and potentiating effect of hypoxic conditions.

## Supplementary Materials

The following are available online at http://www.mdpi.com/2072-6694/10/4/126/s1, Figure S1: Cells were treated for 72 h with vehicle, CDDP, APR-246 or CDDP/APR-246. AnnexinV/PI flowcytometric assay presented as dotplot in four quadrants (AnnV-/PI-; AnnV+/PI-; AnnV+/PI+ and AnnV-/PI+) under normoxic (21% O_2_) and hypoxic (1% O_2_) conditions, Figure S2: HIF-1α and p53 protein levels in NCI-H2228^Q331^* in response to increasing exposure times to hypoxic conditions (1% O_2_) determined by western blotting. HIF-1α staining showed two bands at 116 kDa (processed HIF-1α) and 96 kDa (unprocessed HIF-1α). β-actin was used as internal control, Table S1: IC50-values and Average Combination Index, Table S2: Primers RT-qPCR.
