# Supplementary material for: Hypoxia-Induced Cisplatin Resistance in Non-Small Cell Lung Cancer Cells Is Mediated by HIF-1α and Mutant p53 and Can Be Overcome by Induction of Oxidative Stress
Source: Cancers (Basel). 2018 Apr 21;10(4):126. doi: 10.3390/cancers10040126 (PMC5923381; doi:10.3390/cancers10040126)
Supplement: Supplementary file 1 [file cancers-10-00126-s001.zip › SupplementaryFiles/Supplemental_Figures.docx]

Supplemental Figures


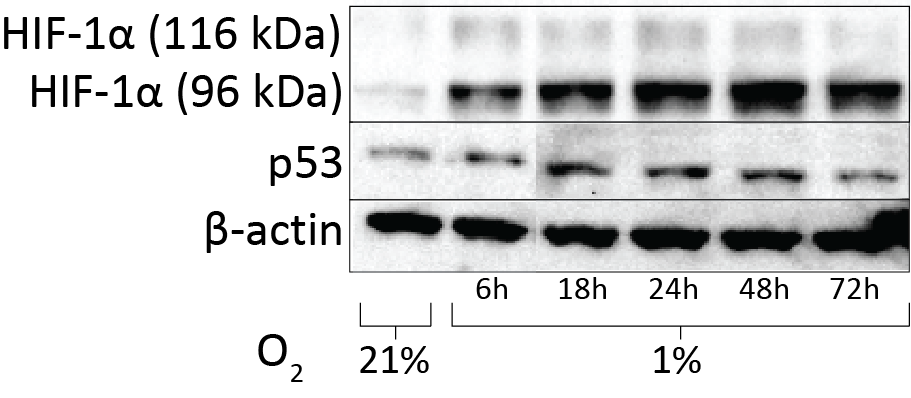


**Figure S1:** HIF-1α and p53 protein levels in NCI-H2228^Q331*^ in response to increasing exposure times to hypoxic conditions (1% O_2_) determined by western blotting. HIF-1α staining showed two bands at 116 kDa (processed HIF-1α) and 96 kDa (unprocessed HIF-1α). β-actin was used as internal control.


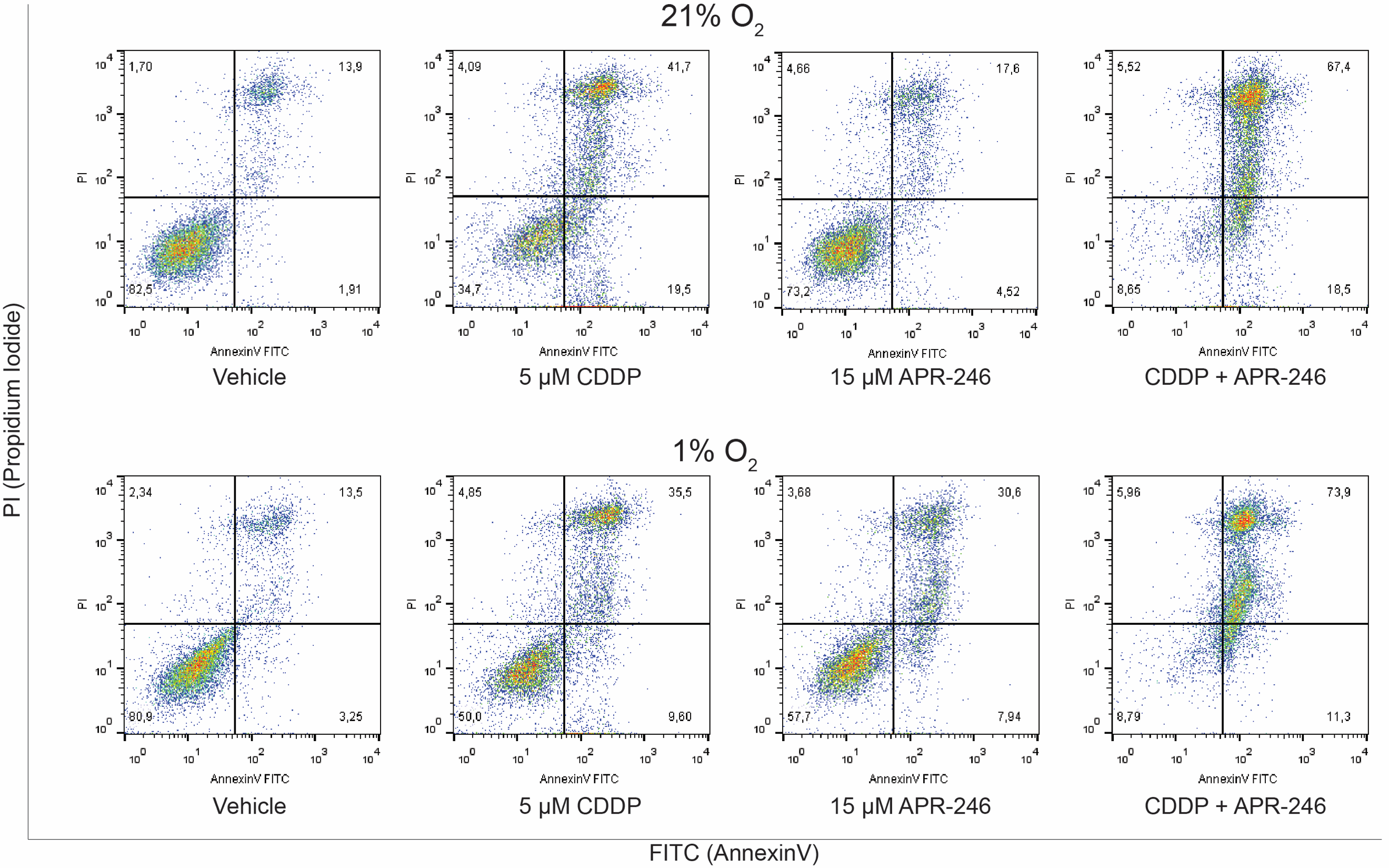


**Figure S2:** Cells were treated for 72h with vehicle, CDDP, APR-246 or CDDP/APR-246. AnnexinV/PI flowcytometric assay presented as dotplot in four quadrants (AnnV-/PI-; AnnV+/PI-; AnnV+/PI+ and AnnV-/PI+) under normoxic (21% O_2_) and hypoxic (1% O_2_) conditions.
