# Supplementary material for: Hypoxia-Induced Cisplatin Resistance in Non-Small Cell Lung Cancer Cells Is Mediated by HIF-1α and Mutant p53 and Can Be Overcome by Induction of Oxidative Stress
Source: Cancers (Basel). 2018 Apr 21;10(4):126. doi: 10.3390/cancers10040126 (PMC5923381; doi:10.3390/cancers10040126)
Supplement: Supplementary file 1 [file cancers-10-00126-s001.zip › SupplementaryFiles/Supplemental_Tables.docx]

| **Table S1**: Primers RT-qPCR | | | |
| --- | --- | --- | --- |
| Target Gene | Target Type | Sequence (5' to 3') | Amplicon Length |
| MDM2 | p53 TT | F-TGGCGTGCCAAGCTTCTCTGT | 100 |
|  |  | R-ACCTGAGTCCGATGATTCCTGCTG |  |
| p21 | p53 TT | F-GGCAGACCAGCATGACAGATTTC | 63 |
|  |  | R-GCTTCCTCTTGGAGAAGATCAGC |  |
| PUMA | p53 TT | F-CCTGGAGGGTCCTGTACAATCT | 88 |
|  |  | R-GCACCTAATTGGGCTCCATCT |  |
| BAX | p53 TT | F-GCTGTTGGGCTGGATCCAAG | 139 |
|  |  | R-TCAGCCCATCTTCTTCCAGA |  |
| NOXA | p53 TT | F-TGTGTTCCTGTTGGGCGTTA | 95 |
|  |  | R-TACTGGCCCCAAGTAACCCT |  |
| CA9 | HIF-1 TT | F-AAATCGCTGAGGAAGGCTCA | 85 |
|  |  | R-TGGAAGTAGCGGCTGAAGTC |  |
| GAPDH | HIF-1 TT | F-TGCACCACCAACTGCTTAGC | 90 |
|  |  | R-GGCATGGACTGTGGTCATGAG |  |
| BNIP3 | HIF-1 TT | F-TGGACGGAGTAGCTCCAAGA | 131 |
|  |  | R-CTTCCTCAGACTGTGAGCTGT |  |
| GLUT1 | HIF-1 TT | F-TGGCATCAACGCTGTCTTCT | 83 |
|  |  | R-AGCCAATGGTGGCATACACA |  |
| VEGFA | HIF-1 TT | F-AGGAGGAGGGCAGAATCATCA | 76 |
|  |  | R-CTCGATTGGATGGCAGTAGCT |  |
| B2M | HKG | F-TGCTGTCTCCATGTTTGATGTATCT | 86 |
|  |  | R-TCTCTGCTCCCCACCTCTAAGT |  |
| HPRT1 | HKG | F-TGACACTGGCAAAACAATGCA | 94 |
|  |  | R-GGTCCTTTTCACCAGCAAGCT |  |
| SDHA1 | HKG | F-ACTCAGCATGCAGAAGTCAATGC | 70 |
|  |  | R-ACCTTCTTGCAACACGCTTCCC |  |
| HMBS | HKG | F-GGCAATGCGGCTGCAA | 64 |
|  |  | R-GGGTACCCACGCGAATCAC |  |
| GADPH | HKG | F-TGCACCACCAACTGCTTAGC | 90 |
|  |  | R-GGCATGGACTGTGGTCATGAG |  |
| YWHAZ | HKG | F-ACTTTTGGTACATTGTGGCTTCAA | 93 |
|  |  | R-CCGCCAGGACAAACCAGTAT |  |
| PMM1 | HKG | F-GCTTCGACACCATCCACTTCTTTG | 63 |
|  |  | R-AGATCTCAAAGTCGTTCCCACCAG |  |
| RPL13A | HKG | F-CCTGGAGGAGAAGAGGAAAGAGA | 125 |
|  |  | R-TTGAGGACCTCTGTGTATTTGTCAA |  |
| p53 TT: p53 transcription target; HKG: housekeeping gene | | | |

| **Table S2: IC50-values and Average Combination Index** | | | | | | |
| --- | --- | --- | --- | --- | --- | --- |
| **O_2_** | **Treatment** | **NCI-H2228** | | | | |
|  |  | **IC_50_ (µM)** | **StDev** | **p-value*** | **CI** | **StDev** |
| **21%** | **APR-246** | 19.88 | 3.76 | / | / | / |
|  | **CDDP** | 2.61 | 0.59 | / | / | / |
|  | **CDDP + 15 μM APR-246** | 1.48 | 0.20 | **0.021** | 0.611 | 0.381 |
|  | **CDDP + 20 μM APR-246** | 1.39 | 0.08 | **0.015** | 0.663 | 0.307 |
| **1%** | **APR-246** | 21.04 | 2.10 | / | / | / |
|  | **CDDP** | 9.49 | 2.07 | / | / | / |
|  | **CDDP + 15 μM APR-246** | 2.35 | 0.29 | **0.001** | 0.596 | 0.291 |
|  | **CDDP + 20 μM APR-246** | 3.17 | 0.82 | **0.002** | 0.611 | 0.358 |
| **O_2_** | **Treatment** | **NCI-H1975** | | | | |
|  |  | **IC_50_ (µM)** | **StDev** | **p-value*** | **CI** | **StDev** |
| **21%** | **APR-246** | 12.03 | 3.07 | / | / | / |
|  | **CDDP** | 8.31 | 1.48 | / | / | / |
|  | **CDDP + 7.5 μM APR-246** | 5.57 | 1.26 | 0.084 | 0.832 | 0.222 |
|  | **CDDP + 10 μM APR-246** | 4.25 | 1.00 | **0.018** | 0.743 | 0.290 |
| **1%** | **APR-246** | 11.99 | 3.35 | / | / | / |
|  | **CDDP** | 10.62 | 1.93 | / | / | / |
|  | **CDDP + 7.5 μM APR-246** | 7.33 | 1.91 | 0.065 | 0.837 | 0.192 |
|  | **CDDP + 10 μM APR-246** | 5.83 | 1.44 | **0.010** | 0.818 | 0.253 |
| **O_2_** | **Treatment** | **A549** | | | | |
|  |  | **IC_50_ (µM)** | **StDev** | **p-value*** | **CI** | **StDev** |
| **21%** | **APR-246** | 24.33 | 2.04 | / | / | / |
|  | **CDDP** | 4.12 | 0.50 | / | / | / |
|  | **CDDP + 20 μM APR-246** | 5.06 | 1.92 | 0.500 | 0.920 | 0.184 |
|  | **CDDP + 25 μM APR-246** | 3.95 | 0.81 | 0.976 | 0.832 | 0.313 |
| **1%** | **APR-246** | 17.45 | 1.09 | / | / | / |
|  | **CDDP** | 4.67 | 0.58 | / | / | / |
|  | **CDDP + 20 μM APR-246** | 4.80 | 0.72 | 0.972 | 0.915 | 0.136 |
|  | **CDDP + 25 μM APR-246** | 5.52 | 1.23 | 0.377 | 1.085 | 0.417 |
| CDDP: Cisplatin; StDev: Standard Deviation of at least 3 independent experiments; CI: Combination Index. *p < 0.05 indicates a significant reduction in CDDP IC_50_ value. | | | | | | |
|  |  |  |  |  |  |  |
